# High temperatures on mental health: Recognizing the association and the need for proactive strategies—A perspective

**DOI:** 10.1002/hsr2.1729

**Published:** 2023-12-04

**Authors:** Moustaq Karim Khan Rony, Hasnat M. Alamgir

**Affiliations:** ^1^ Department of Public Health Bangladesh Open University Gazipur Bangladesh; ^2^ Department of Institute of Social Welfare and Research University of Dhaka Dhaka Bangladesh; ^3^ Department of Career & Professional Development Services (CPDS) Southeast University Dhaka Bangladesh

**Keywords:** heat stress, high temperature, mental health, proactive strategies

## Abstract

**Background and Aims:**

The influence of temperature on various aspects of daily life is often underestimated, and its effects on mental health are not widely recognized. Understanding and addressing the relationship between temperature and mental well‐being is crucial in the context of climate change and rising global temperatures. This perspective aimed to investigate the effects of high temperatures on mental health and identify proactive strategies to mitigate these effects.

**Methods:**

This perspective adopted a twofold approach, including a comprehensive literature review and socioecological framework. The literature review involved extensive searches across Google Scholar, PubMed, and Scopus to identify relevant, peer‐reviewed articles, and reports from diverse disciplines.

**Results:**

The perspective emphasized the significance of recognizing heat stress and its consequences on mental well‐being. Chronic heat stress can lead to increased stress, anxiety, and cognitive impairment. Vulnerable populations include, the very young, older adults, and individuals with pre‐existing mental health conditions. Socioeconomic factors can further exacerbate vulnerability, highlighting the need for tailored strategies to manage mental health challenges during high temperatures. Additionally, the article identified and discussed proactive coping strategies to minimize both the psychological and physical impacts of heat stress. Mindfulness, stress management techniques, and therapy are suggested as effective means for individuals to manage psychological distress.

**Conclusion:**

Implementing preventive measures are essential steps in promoting mental wellness in high temperatures. Proactive strategies by addressing the physiological and psychological effects of heat and considering the specific needs of vulnerable populations can help individuals and communities navigate the challenges posed by rising temperatures and promote resilience and preserve their mental well‐being.

## 1 INTRODUCTION

In an era defined by rapid and visible climate change, the intricate relationship between environmental shifts and human health has come under intense scrutiny.^1^, ^2^ Among many concerns, the impact of rising temperatures on mental health has emerged as a compelling yet often overlooked and under investigated facet.^3^, ^4^ As global temperatures soar to unprecedented levels, its impact on physiological and psychological health is becoming a serious health issue the influence of extreme heat on mental well‐being is assumed/thought to be profound.^5^, ^6^ This burgeoning field of research is shedding light on the multifaceted ways in which rising temperatures can erode the delicate balance of the mind and a comprehensive understanding of the issue is directly needed.^7^

At its core, the association between high temperatures and mental health rests upon a complex interplay of biological and psychological responses.^8^, ^9^ Prolonged exposure to elevated temperatures triggers a cascade of physiological reactions, ranging from accelerated heart rates and increased sweating leading to dehydration.^10^, ^11^ These responses, while crucial for maintaining thermal equilibrium, can exert subtle yet important effects on mental health. Studies have unveiled correlation between prolonged heat exposure and heightened levels of stress and irritability.^12^ The very physical discomfort induced by heat stress can create a breeding ground for psychological distress, a sentiment exacerbated by the disruption of sleep patterns—a common consequence of heat‐related discomfort and this leads to fatigue and cognitive impairments.^13^

The impact that temperature plays on human lives is crucial yet frequently undervalued. It affects many daily decisions, and the activities people engage each day, with impacts that extend far beyond listening to and acting on the weather forecast.^14^ For instance, choice of clothing is decided by it.^15^ It also influences what people eat and drink; a hot summer day may call for a refreshing salad and iced tea, while a chilly winter's night may inspire people to have stew and cocoa.^16^ The sports, hobbies, and even the vacation decisions and destinations are selected by the prevailing temperature.^17^

Furthermore, temperature influences physical health. Human bodies constantly work to maintain a stable internal temperature, and extremes on either end of the temperature spectrum can place stress on this regulatory system.^18^ Long periods in freezing temperatures can cause hypothermia and frostbite, whereas long periods in high heat can cause heat exhaustion, and even heatstroke.^19^ Heatwaves have been linked to increases in mood disorders and anxiety.^20^ On the other hand, the colder weather and less sunlight during the winter can exacerbate seasonal affective disorder (SAD), a depression linked to seasonal changes.^21^ However, since the world now faces increasing temperatures due to climate change, understanding the profound effects of heat on mental wellbeing becomes increasingly important. This perspective aimed to explore the possible impact of elevated temperatures on mental well‐being and identify and list proactive measures for alleviating some of the consequences.

### 1.1 The heat‐mental health connection

High temperatures have a direct and noticeable effect on people's health. From mild discomfort to life‐threatening conditions, the physiological repercussions of heat are extensive and multifaceted.^22^ Dehydration, which occurs when one loses more fluids than he/she takes in, disrupts the normal functioning of the body's systems and is the most fundamental way heat impacts human bodies.^23^ Mild symptoms include thirst and a dry mouth, whereas more severe symptoms include disorientation, confusion, and even coma.^24^ However, the risks of high temperatures do not stop at dehydration. Heat exhaustion is another common heat‐related illness. It occurs when the body's internal temperature rises above its normal range, usually due to strenuous activity in hot weather or continuous exposure to high temperatures.^25^ Without proper intervention, heat exhaustion can progress to heatstroke.^26^

Heatstroke is the most severe form of heat injury and is considered a medical emergency. Damage to the brain, heart, kidneys, and muscles may occur if the body temperature increases to 104°F (40°C) or higher.^11^ It is often the culmination of the unchecked progression of other heat‐related illnesses and requires immediate medical attention to prevent serious complications or death. However, it is crucial to understand that these physical health impacts do not exist in isolation from the mental wellbeing. The body‐mind connection is fundamental to health, and physical discomfort or illness often has psychological implications. For instance, fatigue and discomfort from dehydration or heat exhaustion can lead to increased irritability and concentration difficulties.^27^ In more severe cases, the confusion associated with heatstroke can be accompanied by agitation, changes in behavior, and even hallucinations.^28^

The relationship between heat and mental health is not merely anecdotal or intuitive but is grounded in a growing body of scientific research. Several studies have been conducted in recent years that underscore this connection. One study found that heatwaves significantly increase the risk of hospital admissions for mental and behavioral disorders.^29^ Aguglia et al. found that the likelihood of hospitalization for mood disorders like depression and mania increased by approximately 40% during periods of high heat.^30^ Another study demonstrated a notable correlation between high temperatures and increased suicide rates.^31^ In addition, a study by Hu et al. found that suicide rates rose by 1% for each 1°C increase on average monthly temperature.^32^ Yet another study found that deviations from moderate temperatures and precipitation patterns systematically increase the risk of conflict, whether interpersonal, such as rape and domestic violence, or intergroup, like civil wars.^33^, ^34^ These studies serve to emphasize the importance of acknowledging and addressing the effect of heat on mental health. With global temperatures projected to continue rising due to climate change, the relevance and urgency of this topic have become more important. Therefore, understanding this connection is the first step toward creating strategies that will allow bear the heat while maintaining mental wellness.

## 2 METHODS

In this context, a comprehensive investigation into the impact of high temperatures on mental health was conducted using a twofold approach: a thorough literature review and socioecological framework.

### 2.1 Literature review

To understand the relationship between high temperatures and mental health, an exhaustive review of existing literature was performed. Academic databases, including Google Scholar, PubMed, and Scopus, were extensively searched to identify relevant studies and peer‐reviewed articles that have explored the subject matter. The literature review encompassed a diverse range of disciplines, such as environmental science, psychology, public health, and climate studies, to offer a multidisciplinary perspective on the issue. By drawing insights from various sources, the literature review aimed to synthesized and present a comprehensive and evidence‐based overview of the topic.

### 2.2 Socioecological framework

The socioecological framework draws from diverse disciplines—including psychology, environmental science, public health—to comprehensively explore the intricate relationship between temperature, vulnerability, resilience, and mental well‐being.^35^, ^36^, ^37^ It underscores the importance of acknowledging heat stress, considering socioecological contexts, and implementing proactive measures to foster mental wellness in the context of escalating global temperatures.^38^ This framework recognizes that temperature, a fundamental yet often overlooked environmental factor, profoundly affects mental well‐being.^39^

Guided by the premise that temperature acts as an environmental stressor, this framework delves into how it greatly impacts cognitive and emotional states, thereby influencing mental health outcomes.^36^ The Transactional Model of Stress and Coping forms a foundational pillar, elucidating how individuals perceive and navigate heat stress through cognitive appraisals and coping mechanisms.^35^ Through this lens, the framework reveals the cognitive strain induced by high temperatures, which can amplify discomfort, elevate stress levels, and contribute to the emergence of mental health challenges.^40^

The socioecological dimension of the framework extends its reach to vulnerable populations. Vulnerability factors, such as age, pre‐existing mental health conditions, and socioeconomic disparities, are explored to highlight the heightened susceptibility of specific groups to heat‐induced mental health issues.^37^ Resilience factors like social support networks, adaptive coping strategies, and community cohesion emerge, mitigating the adverse impact of high temperatures on mental well‐being.^41^ Additionally, the framework integrates insights from eco‐psychology, emphasizing the potential of nature‐based interventions to alleviate psychological distress caused by heat stress.^42^ By considering the biophilic connection between individuals and the natural environment, the framework proposes that exposure to green spaces and therapeutic interventions can contribute to mental wellness.^38^

Within this theoretical backdrop, the article underscores the significance of proactive strategies. It emphasizes the necessity for tailored interventions addressing both the physiological and psychological effects of high temperatures.^36^ By incorporating mindfulness, stress management techniques, and therapy as proactive coping mechanisms, the framework promotes individual and community resilience against the mental health challenges posed by rising temperatures.^39^

## 3 HEAT STRESS AND ITS CONSEQUENCES

Heat stress exerts a profound impact on the cardiovascular system and blood flow. Elevated temperatures trigger vasodilation, widening blood vessels to dissipate heat, which can lead to reduced blood pressure and compromised circulation.^26^ The heart works harder to maintain blood flow, potentially causing strain. Dehydration from excessive sweating further thickens the blood, increasing clotting risks.^43^ Heat stress prompts increased heart rate, elevating demand on the heart. Prolonged exposure may contribute to endothelial dysfunction, impairing blood vessel health. Such intricate interplay underscores the importance of managing heat stress to safeguard cardiovascular well‐being and maintain optimal blood flow.^44^

Heat stress refers to the body's inability to regulate its internal temperature and cool itself down.^44^ This typically results from prolonged exposure to hot temperatures, particularly when strenuous physical activity is involved. This condition is significant as it not only poses potential physical health risks like heat exhaustion or heatstroke, but it can also adversely impact an individual's mental health.^11^ High temperatures can increase discomfort, interfere with sleep, and alter daily routines, potentially leading to an escalation in stress, anxiety, and even cognitive impairment if unattended.^45^ These physical symptoms can have serious implications for mental health. Disrupted sleep patterns due to heat can contribute to mood fluctuations and exacerbate mental health conditions like depression and anxiety.^20^ In addition, psychosis, hallucinations, and other neuropsychiatric symptoms may occur in people with severe heatstroke.^46^

Chronic heat stress, sustained over a long period, can have profound effects on mental wellness. Persistent exposure to high temperatures, along with the related physical discomfort and sleep disruptions, can lead to increased levels of stress and anxiety.^12^ Furthermore, these factors may exacerbate pre‐existing mental health illnesses or cause new ones to emerge. Long‐term exposure to high temperatures can also affect cognitive abilities like recall, concentration, and intellectual ability.^47^ Therefore, proactive strategies for managing heat stress can play a crucial role in promoting overall mental wellness.

### 3.1 Vulnerable populations

Age‐related heat vulnerabilities are a significant concern, as both the very young and older adults have a heightened risk of heat‐related illness.^11^ Children, especially infants, possess a lesser ability to regulate their body temperature and thus can quickly become overheated.^48^ On the other hand, older adults have diminished physiological mechanisms to respond effectively to heat.^22^ Heart disease, obesity, and diabetes are more common in older adults and can further impair the body's capacity to cool down.^49^ Moreover, cognitive impairments or mobility issues can prevent older individuals from recognizing the signs of heat stress or taking necessary actions.^49^ Thus, age‐related factors significantly increase vulnerability to heat stress and related mental health problems. Likewise, psychotropic medications can impede the body's heat regulation, leading to increased susceptibility to heat stress.^50^ In severe cases, heat stress can cause confusion and delirium, potentially exacerbating cognitive impairment in individuals with schizophrenia or other cognitive disorders.^51^ This makes the summer months potentially challenging for those with mental health conditions and necessitates tailored strategies to manage their wellbeing effectively.

Furthermore, socioeconomic factors play a crucial role in heat vulnerability. Lower‐income individuals often lack access to cooling measures like air conditioning, making them more prone to heat stress.^25^ Occupations involving strenuous outdoor work or those lacking adequate cooling facilities, which are often lower‐wage jobs, can lead to increased heat exposure. Additionally, neighborhoods with less greenery and more concrete, common in lower‐income areas, can be hotter due to the urban heat island effect. Limited access to healthcare and lack of knowledge about heat stress can lead to delayed treatment in these populations. Therefore, it is essential that heat mitigation strategies take these socioeconomic factors into account, ensuring that interventions reach those most vulnerable to the effects of high temperatures.

## 4 COPING STRATEGIES—THE PSYCHOLOGICAL APPROACH

Understanding and recognizing heat stress is critical for managing its effects on mental health. Heat stress occurs when the body cannot adequately cool itself and typically results from prolonged exposure to high temperatures.^44^ Psychological symptoms can include stress, irritability, sleep disturbances, reduced motivation, decreased mood and enjoyment, and agitation (Table 1).^60^ However, mindfulness and stress management techniques can be effective in mitigating the mental health impact of heat stress. Mindfulness involves staying present and calmly acknowledging one's feelings, thoughts, and bodily sensations.^61^ This approach can help individuals manage their reactions to heat, thus reducing psychological distress. Techniques may include mindful breathing exercises, guided meditations, or progressive muscle relaxation. Stress management strategies such as maintaining a regular sleep schedule, eating a balanced diet, staying hydrated, and taking breaks from heat exposure can also be beneficial.^62^

**Table 1:** Psychological response to heat.

Psychological response	Explanation
Increased anxiety	High temperatures can induce feelings of anxiety and unease. The body's stress response is activated, leading to heightened levels of stress hormones, such as cortisol and adrenaline. These physiological changes can contribute to a sense of restlessness and apprehension, intensifying anxiety symptoms.^52^
Irritability and agitation	Heat stress triggers the release of stress hormones, potentially leading to increased irritability and agitation. The physiological stress response to high temperatures includes the release of stress hormones like adrenaline and cortisol. These hormones can evoke emotional responses, leading to feelings of irritability, restlessness, and heightened emotional sensitivity.^53^ Individuals may find themselves more prone to frustration, impatience, and interpersonal conflicts during heat exposure, underscoring the intricate link between physiological and emotional well‐being.^54^
Mood disturbances	Prolonged exposure to high temperatures can disrupt mood stability. Heat‐induced physiological changes, such as hormonal fluctuations and disturbed sleep patterns, can contribute to shifts in mood. Individuals may experience heightened mood swings, feelings of sadness, or an overall sense of emotional instability.^55^
Reduced patience and tolerance	Prolonged exposure to high temperatures can decrease patience and tolerance levels. The discomfort and stress associated with heat stress can lower the threshold for irritation, leading to reduced patience in interpersonal interactions and heightened emotional sensitivity.^56^
Decreased coping abilities	High temperatures can diminish an individual's ability to cope with stress. The physiological strain of heat stress can weaken psychological resilience, making it harder to manage daily stressors and challenges effectively. This can lead to a heightened perception of stress and a diminished sense of control over one's emotions.^22^
Sleep disruption	Elevated temperatures can disturb sleep patterns, impacting mental well‐being. Poor sleep quality due to heat can lead to restlessness, difficulty falling asleep, and frequent awakenings during the night. Sleep disruption can exacerbate existing mental health conditions and contribute to feelings of irritability and fatigue.^57^
Decreased motivation	Heat‐induced discomfort may lead to decreased motivation to engage in activities. The physiological strain of high temperatures can drain energy levels and dampen enthusiasm, potentially resulting in reduced participation in social, work, and leisure activities.^58^
Feelings of helplessness	Prolonged exposure to extreme heat can evoke a sense of helplessness and vulnerability. The perception of being unable to escape or control the environmental stressor can lead to feelings of despair and powerlessness, contributing to emotional distress.^45^
Aggravation of pre‐existing mental health conditions	High temperatures can exacerbate symptoms of pre‐existing mental health conditions. Individuals with conditions such as anxiety disorders, depression, or bipolar disorder may experience intensified symptoms during heatwaves, making it crucial to manage their mental health effectively.^59^

Therapy and counseling options can play a vital role in coping with heat‐induced mental stress. Individuals may acquire the ability to recognize and alter destructive patterns of thought and action through cognitive behavioral therapy (CBT).^63^ For those experiencing severe anxiety or depressive symptoms due to heat stress, talking therapies can offer a safe space to explore feelings and develop coping strategies.^64^ In addition, therapists can assist in adapting treatment regimens for patients with pre‐existing mental health disorders who are particularly susceptible to heat.^65^ It is essential to consult with a healthcare provider or a mental health professional to discuss the most appropriate therapy or counseling options.

### 4.1 Coping strategies**—**The physical approach

A robust body of research underscores the pivotal role that proper hydration and nutrition play in upholding physical well‐being during periods of heightened heat. A study by Périard et al. highlighted the detrimental impact of dehydration on the physical manifestations of heat stress, leading to heightened discomfort, augmented fatigue, and subsequently, an exacerbation of psychological distress (Table 2).^44^ Consequently, it is imperative to prioritize the consistent consumption of water or hydrating fluids, irrespective of thirst cues, to ensure optimal physiological functioning.

**Table 2:** Physiological stress response to heat.

Physiological response	Explanation
Increased heart rate	High temperatures cause blood vessels to dilate, leading to an increased heart rate as the body attempts to cool down. This heightened cardiovascular activity is part of the body's thermoregulation mechanism, aiming to redistribute heat and maintain internal temperature equilibrium.^66^ The increased heart rate is intended to enhance blood flow to the skin's surface, facilitating heat dissipation through sweating.^67^ However, prolonged elevated heart rate due to heat stress can strain the cardiovascular system over time.^68^
Increased respiration rate	Heat stress can elevate respiration rate as the body attempts to release excess heat. Faster and deeper breathing facilitates heat exchange by expelling warm air and taking in cooler air, aiding temperature regulation.^69^, ^70^
Dehydration	Heat accelerates fluid loss through sweating, potentially leading to dehydration and affecting cognitive function. Sweating is the body's primary cooling mechanism during heat exposure.^71^ As sweat evaporates, it carries away excess heat, but this process also results in the loss of vital fluids and electrolytes.^72^ Dehydration can impair blood circulation, decrease cognitive performance, and intensify feelings of fatigue and irritability. Severe dehydration can escalate into a medical emergency, warranting immediate attention and fluid replacement.^73^
Vasodilation	Blood vessels expand to dissipate heat, diverting blood flow from vital organs, which can impact cognitive and physical performance. Vasodilation is the body's response to elevated temperatures, designed to direct blood toward the skin's surface for heat dissipation.^74^ However, this redirection can lead to reduced blood supply to essential organs, potentially affecting their optimal function. This diversion of blood flow can contribute to decreased cognitive performance, impaired decision‐making, and reduced physical capabilities, ultimately impacting overall well‐being.^75^
Electrolyte imbalance	Excessive sweating can disrupt electrolyte balance, affecting nerve and muscle function and contribute to fatigue. Sweating not only results in fluid loss but also leads to the depletion of essential electrolytes, such as sodium, potassium, and chloride.^76^ Electrolytes play a crucial role in maintaining proper nerve conduction and muscle function. Imbalances can lead to muscle cramps, weakness, and cognitive disturbances. Addressing electrolyte imbalances becomes crucial to mitigate fatigue, support cognitive clarity, and maintain overall physiological stability.^77^
Cognitive impairment	Heat‐induced stress can impair cognitive function, affecting memory, attention, and decision‐making abilities. Prolonged exposure to high temperatures can trigger a stress response in the body, leading to the release of stress hormones such as cortisol.^78^ Elevated cortisol levels can impair cognitive processes, including memory formation, attention span, and complex problem‐solving. Heat‐induced cognitive impairments can hinder daily tasks, exacerbate stress, and contribute to a sense of mental strain and frustration.^79^
Increased perceived exertion	Physical tasks may be felt more challenging in hot conditions, leading to a perception of increased effort and fatigue. High temperatures can impose additional strain on the body during physical activities.^80^ As the body works to dissipate heat and maintain internal temperature, individuals may perceive physical tasks as more demanding than usual. This increased perception of effort can lead to feelings of fatigue and exhaustion, limiting the motivation and ability to engage in daily activities and potentially contributing to decreased overall physical and mental well‐being.^78^
Enhanced risk of heat‐related illnesses	Heat exhaustion and heatstroke can result from prolonged exposure, causing confusion, disorientation, and anxiety. Prolonged exposure to high temperatures without proper mitigation measures can elevate the risk of heat‐related illnesses.^81^ Heat exhaustion is characterized by symptoms such as confusion, dizziness, nausea, and weakness. Left untreated, heat exhaustion can escalate to heatstroke, a life‐threatening condition marked by elevated body temperature, confusion, disorientation, and potential damage to internal organs. The onset of these heat‐related illnesses can trigger anxiety and distress, further highlighting the significant implications of high temperatures on mental health.^82^

The integration of fruits and vegetables into one's diet emerges as a paramount strategy, backed by a wealth of research, for supplying vital vitamins and minerals that facilitate the maintenance of an optimal body temperature and overall functionality.^83^ Furthermore, scientific investigations emphasize the benefits of eschewing heavy meals, which have been shown to contribute to escalated metabolic heat and subsequent body warmth.^84^

About mental well‐being, a plethora of studies underline the indispensability of rest and sleep in navigating high temperatures without compromising mental equilibrium. Elevated temperatures have been repeatedly found to be associated with an amplification of stress, mood fluctuations, and an exacerbation of prevailing mental health challenges.^85^ Counteracting these effects, research underscores the effectiveness of creating a cool sleeping environment through mechanisms such as fans, air conditioning, or presleep cooler showers, all of which have been shown to enhance the quality of sleep.^86^ Notably, periods of rest during the most sweltering segments of the day have been established as instrumental in mitigating the toll of heat‐induced physical and mental exhaustion.^49^

Many studies continue to emphasize the significance of appropriate clothing choices and environmental adaptations in ameliorating the effects of heat exposure. Khosla et al. revealed that wearing lightweight, light‐colored, and loose‐fitting clothing can enhance effective body cooling. They specifically recommended the use of breathable materials like cotton or linen.^87^ Augmenting this, investigations advocate for the use of hats and sunglasses for supplementary outdoor protection.^88^ On the environmental front, research substantiates the advantages of maintaining cool indoor spaces through mechanisms such as window shading.^22^ Libraries and community centers, often equipped with air conditioning, have been established as invaluable resources for seeking respite from the heat when access is limited at home.^89^ Additionally, studies highlight the effectiveness of indoor space ventilation during cooler periods of the day to facilitate air circulation and reduce indoor temperatures.^90^ In light of this extensive body of research, it is evident that a holistic approach encompassing hydration, nutrition, rest, clothing, and environmental considerations is imperative for maintaining both physical and mental well‐being amidst elevated temperatures.

## 5 THE ROLE OF COMMUNITY AND POLICY

Community awareness and support are vital components in managing the mental health implications of heat stress. Awareness programs can educate the public about the signs of heat stress, potential mental health impacts, and strategies for staying cool. Moreover, the community can play a supportive role by checking on vulnerable neighbors during heatwaves, such as the elderly, those with pre‐existing conditions, or individuals living alone.^91^ Community centers can also provide cool spaces for those without access to air conditioning while fostering a sense of togetherness during challenging times.

Government and workplace policies can significantly mitigate the effects of heat stress. Governments can enact heat action plans that include issuing warnings for heatwaves, providing public cooling centers, and offering support for vulnerable populations.^92^ Workplace policies should address the needs of employees exposed to high temperatures by offering breaks, cool rest areas, flexible scheduling to avoid the hottest parts of the day, and training to recognize the symptoms of heat stress. It is also essential to ensure access to mental health resources in the workplace to address any mental health implications of heat stress.

### 5.1 Resilience, advocacy, and call to action

Each of us has the potential for resiliency in the face of heat‐related issues, and this fact should not be overlooked. By staying informed, taking care of one's mental and physical health, and extending the support to others, resilience can be built. Furthermore, advocacy plays a crucial role in addressing heat stress on a broader scale. Advocacy efforts can aim at driving policy changes that promote heat‐resistant urban design, such as increasing green spaces, and ensuring affordable access to cooling solutions.

Heatwaves are expected to grow more often and intensely as climate change continues. This situation calls for a proactive approach to managing the mental health implications of heat stress. A call to action could involve steps like advocating for mental health considerations in heat action plans, supporting heat‐vulnerable populations, and pushing for changes in urban design to mitigate heat. Therefore, climate change is now seen as an environmental problem and a public health crisis that requires an urgent response.

## 6 STRENGTHS AND LIMITATIONS OF THE STUDY

The article underscores that susceptible populations, encompassing the young, elderly, and individuals with pre‐existing mental health conditions, bear disproportionate effects of heat stress. This recognition accentuates the imperative for precisely targeted interventions. Furthermore, the integration of proactive coping strategies enhances the research's practicality. By proposing mindfulness, heat stress management techniques, and therapy as viable avenues, the article provides tangible mechanisms for individuals to alleviate psychological distress amid rising temperatures.

However, the perspective's reliance on existing literature could potentially introduce bias or limitations intrinsic to the chosen sources. Overreliance on specific disciplines or geographic regions might impinge upon the comprehensive nature of the findings. While the synthesized conceptual model from the literature review holds value, its robustness and applicability could be bolstered through supplementary validation or empirical testing. Additionally, the article predominantly centers on the influence of elevated temperatures on mental health, potentially overshadowing other climate‐related variables that could also contribute to challenges in mental well‐being. Encompassing a broader spectrum of climate factors would yield a more inclusive understanding. Furthermore, the effectiveness and feasibility of the recommended coping strategies could manifest diversely across varying cultural, social, and economic contexts. To enhance the applicability of the proposed strategies, due consideration of these variables is imperative.

## 7 CONCLUSION

Proactive strategies for mental wellness in high temperatures are crucial for coping with the challenges posed by extreme heat. High temperatures can have severe effects on mental health. Consequently, it is essential to take precautions to avoid adverse outcomes. Prioritizing self‐care, such as staying hydrated, seeking shade, and practicing relaxation techniques, is critical to managing mental health during hot weather conditions. Fostering social connections and support networks can provide vital emotional support and a sense of community. Raising awareness about the mental health risks associated with high temperatures is also important. Educating individuals about the signs of heat‐related mental distress and providing resources for support can empower them to take proactive steps to protect their mental well‐being. Implementing these effective strategies at both individual and community levels can enhance mental resilience in high temperatures and promote overall mental wellness.

## 8 RECOMMENDATIONS

As global temperatures continue to rise, it is imperative that forthcoming research studies delve into the long‐term effects, encompassing prolonged heatwaves and their psychological implications, particularly for the vulnerable populations. This research has the potential to lay the foundation for targeted interventions and coping mechanisms. Furthermore, future policy trajectories must distinctly prioritize the mental health consequences of heat stress, recognizing the intricate interplay between climate change, mental well‐being, and social inequity. This entails allocating resources for research into the mental health impacts of heat stress, integrating mental health considerations into climate adaptation strategies, and ensuring that mental health services remain accessible and well‐prepared to address the increased stressors associated with escalating temperatures.^93^

Governments hold the responsibility of weaving mental health considerations into their climate change adaptation blueprints, acknowledging the symbiotic relationship between physical and psychological welfare. Timely interventions to support mental health during heat‐related crises could be facilitated through early warning systems for extreme heat events.

Public education campaigns play a pivotal role in heightening awareness about the mental health risks posed by elevated temperatures, nurturing a culture of adaptive coping. Additionally, communities must be fortified with resources to bolster resilience, including provisions like heat shelters and robust community support networks. Moreover, urban planning should prioritize heat‐resilient designs, encompassing elements such as shaded areas, verdant spaces, and heat‐reflective materials. Collaborative endeavors uniting architects, urban planners, psychologists, and climatologists have the potential to yield groundbreaking urban environments that holistically enhance mental well‐being.

## AUTHOR CONTRIBUTIONS

**Moustaq Karim Khan Rony**: Conceptualization; data curation; formal analysis; investigation; methodology; project administration; resources; supervision; validation; visualization; writing—original draft; writing—review and editing. **Hasnat M. Alamgir**: Data curation; formal analysis; investigation; methodology; project administration; resources; supervision; validation; visualization; writing—original draft; writing—review and editing.

## CONFLICT OF INTEREST STATEMENT

The authors declare no conflict of interest.

## TRANSPARENCY STATEMENT

The lead author Moustaq Karim Khan Rony affirms that this manuscript is an honest, accurate, and transparent account of the study being reported; that no important aspects of the study have been omitted; and that any discrepancies from the study as planned (and, if relevant, registered) have been explained.

## Data Availability

Data sharing is not applicable to this article as no new data were created or analyzed in this study.
